# p38α deficiency restrains liver regeneration after partial hepatectomy triggering oxidative stress and liver injury

**DOI:** 10.1038/s41598-019-39428-3

**Published:** 2019-03-07

**Authors:** Sergio Rius-Pérez, Ana M. Tormos, Salvador Pérez, Isabela Finamor, Patricia Rada, Ángela M. Valverde, Angel R. Nebreda, Juan Sastre, Raquel Taléns-Visconti

**Affiliations:** 1 Department of Physiology, University of Valencia. Burjassot, Valencia, 46100 Spain; 2 Instituto de Investigaciones Biomédicas Alberto Sols (Centro Mixto CSIC-UAM), Arturo Duperier 4, 28029 Madrid, Spain; 3 Centro de Investigación Biomédica en Red de Diabetes y Enfermedades Metabólicas Asociadas (CIBERdem), ISCIII, 28029 Madrid, Spain; 4 Institute for Research in Biomedicine (IRB Barcelona), Barcelona Institute of Science and Technology, 08028 Barcelona, Spain; 5 ICREA, Pg. Lluís Companys 23, 08010 Barcelona, Spain; 6 Department of Pharmacy and Pharmaceutical Technology and Parasitology, University of Valencia. Burjassot, Valencia, 46100 Spain

## Abstract

p38α MAPK negatively regulates the G_1_/S and G_2_/M cell cycle transitions. However, liver-specific p38α deficiency impairs cytokinesis and reduces hepatocyte proliferation during cirrhosis and aging in mice. In this work, we have studied how p38α down-regulation affects hepatocyte proliferation after partial hepatectomy, focusing on mitotic progression, cytokinesis and oxidative stress. We found that p38α deficiency triggered up-regulation of cyclins A1, B1, B2, and D1 under basal conditions and after hepatectomy. Moreover, p38α-deficient hepatocytes showed enhanced binucleation and increased levels of phospho-histone H3 but impaired phosphorylation of MNK1 after hepatectomy. The recovery of liver mass was transiently delayed in mice with p38α-deficient hepatocytes *vs* wild type mice. We also found that p38α deficiency caused glutathione oxidation in the liver, increased plasma aminotransferases and lactate dehydrogenase activities, and decreased plasma protein levels after hepatectomy. Interestingly, p38α silencing in isolated hepatocytes markedly decreased phospho-MNK1 levels, and silencing of either p38α or Mnk1 enhanced binucleation of hepatocytes in culture. In conclusion, p38α deficiency impairs mitotic progression in hepatocytes and restrains the recovery of liver mass after partial hepatectomy. Our results also indicate that p38α regulates cytokinesis by activating MNK1 and redox modulation.

## Introduction

p38α is a redox sensitive MAPK ubiquitously expressed at high levels in most cell types^1^. It is also activated by environmental and genotoxic stress and plays key roles in the control of cell proliferation, differentiation and survival, as well as in the regulation of the inflammatory response^2–4^.

p38α MAPK regulates the G_1_/S and G_2_/M cell cycle checkpoints prior to DNA synthesis and cell division, respectively^5–7^. Increased proliferation and impaired differentiation have been traditionally considered hallmarks of p38α-deficient cells^6^. Thus, it was reported that mice with p38α-deficient hepatocytes exhibited enhanced hepatocyte proliferation after partial hepatectomy (PHx)^8^ and developed more liver tumours with increased number of proliferative cells^9^. Accordingly, activation of p38 MAPK resulted in growth arrest and inhibition of DNA synthesis in cultured foetal rat hepatocytes^5^. In addition, inhibition of p38 MAPK *in vivo* was sufficient to trigger a marked increase in the number of proliferating hepatocytes^5^. However, paradoxically, we recently found that liver-specific p38α deficiency lowered hepatocyte proliferation and enhanced hepatocyte binucleation due to cytokinesis failure in both biliary cirrhosis and aging^3,10^.

Interestingly, hepatocytes are able to change from polyploid to diploid, and from binucleated to mononucleated with reduced ploidy by a phenomenon called somatic ‘reductive mitoses’, due to multipolar mitotic spindles^11^. In fact, ploidy reversal is a useful tool to enhance hepatocyte proliferation^12^. This prompted us to enquire how hepatocyte proliferation evolves when the brake exerted by p38α in the G_1_/S and G_2_/M cell cycle transitions is released. Thus, our aim was to study hepatocyte proliferation and liver regeneration after partial hepatectomy in mice with p38α-deficient hepatocytes. We also determined whether p38α deficiency leads to oxidative stress after partial hepatectomy.

## Results

### p38α MAPK deficiency releases the brake with cyclins

**Figure 1:**
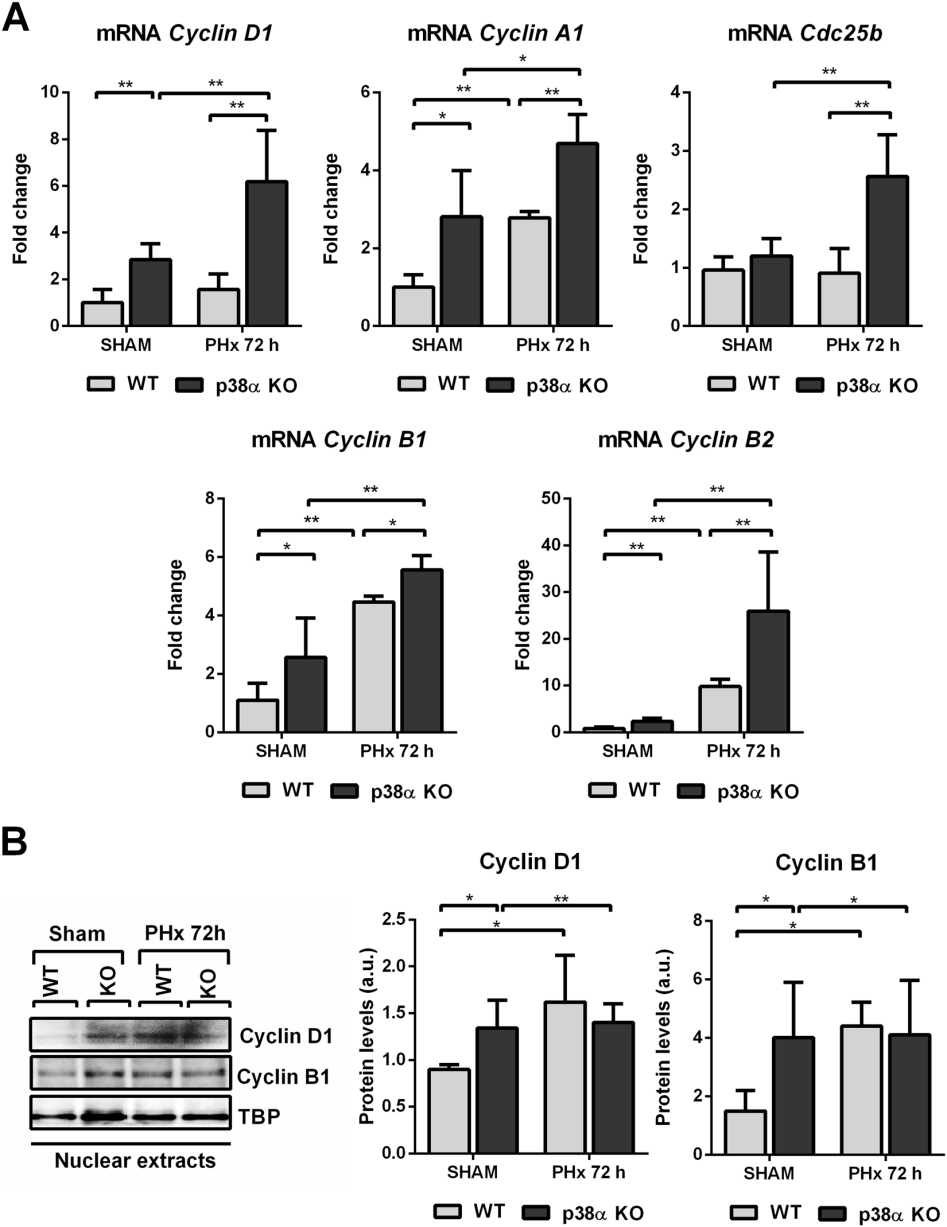
Effect of liver specific p38α MAPK deficiency on cyclin mRNA expression after partial hepatectomy. (**A**) Real-time PCR analysis of the mRNA relative expression for *Cyclin D1*, *A1*, *Cdc25b*, *B1* and *B2* in liver 72 hours after hepatectomy (PHx). Data (mean and SD) are shown as fold increase in mRNA level compared to the control and were normalized by TATA-binding protein mRNA. (**B**) Representative western blot images and densitometries for cyclin D1 and cyclin B1 in nuclear extracts of livers from sham wild type and p38α knockout mice and at 72 hours after PHx. TBP was used as a loading control. *P < 0.05; **P < 0.01. WT, wild type mice; KO, p38α knockout mice; PHx, partial hepatectomy.

p38α is a negative regulator of cyclins, accordingly its deficiency triggered up-regulation of the mRNA of *Cyclins A1*, *B1*, *B2*, and *D1* under basal conditions and after partial hepatectomy, and also induced the expression of *Cdc25b* after PHx (Fig. 1A). The increase in *Cyclin B2* expression in the liver of p38α knockout mice after hepatectomy was remarkable in comparison with wild type mice.

We also performed western blotting of cyclins D1 and B1, which shows that under basal conditions there were indeed increased levels of these cyclins upon p38α deficiency (see Fig. 1B and Supplementary Fig. S1). However, after partial hepatectomy these levels did not further increase and maintained similar levels to those of wild type mice.

### p38α MAPK deficiency triggers cytokinesis failure after partial hepatectomy likely through reduced MNK1 phosphorylation

**Figure 2:**
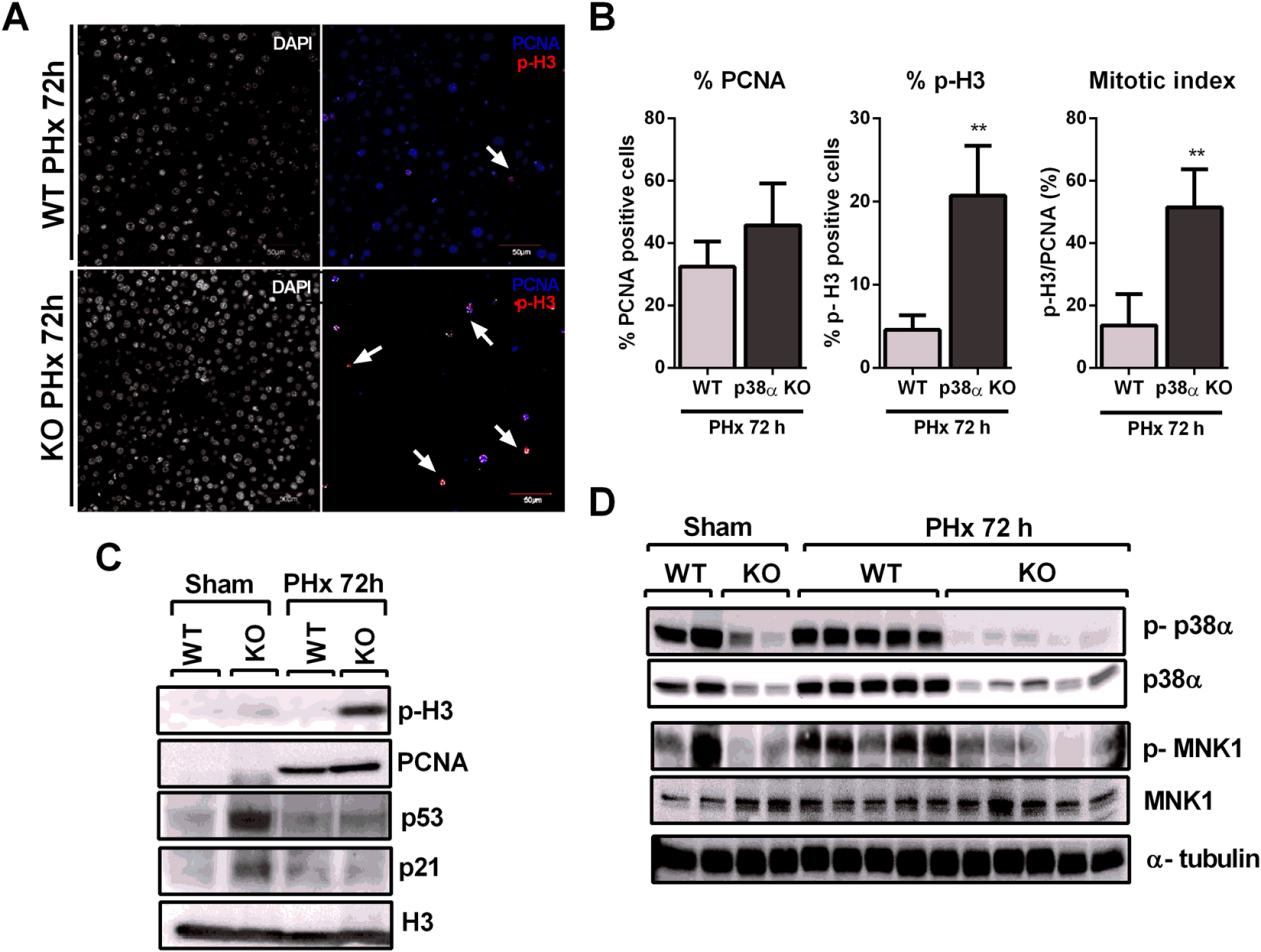
p38α MAPK deficiency reduces MNK1 phosphorylation and triggers cytokinesis failure after partial hepatectomy. (**A**) Representative immunohistochemistry images of liver sections from wild type and liver specific p38α knockout mice after partial hepatectomy. Red, phosphorylated histone 3 (p-H3); blue, PCNA; white, DAPI. A minimum of four experiments were performed for each group of animals. Scale bars = 50 μm. (**B**) Quantification of the percentage of PCNA-positive cells, p-H3-positive cells, and mitotic index expressed as p-H3/PCNA ratio. Data are shown as mean and SD. **P < 0.01. (**C**) Representative western blot images for p-H3, PCNA, p53, p21 and H3 in nuclear extracts of livers from sham wild type and p38α knockout mice and at 72 hours after PHx. H3 was used as loading control. (**D**) Representative western blot for p-p38α, p38α, p-MNK1 and MNK1 from sham and after PHx livers of wild type and p38α knockout mice. α-tubulin was used as loading control. WT wild type mice; KO p38α knockout mice; PHx partial hepatectomy.

**Figure 3:**
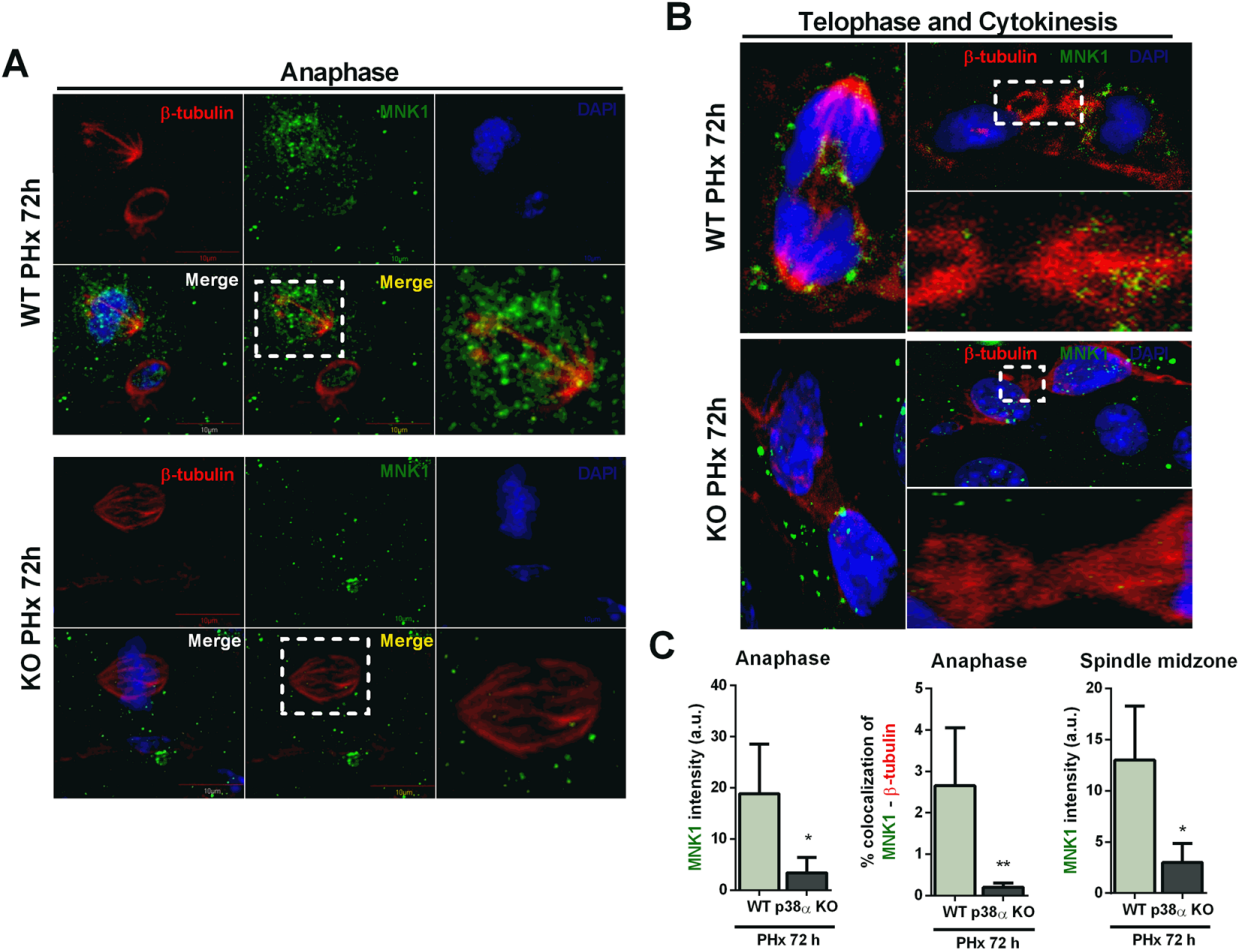
p38α MAPK modulates MNK1 localization during the cell cycle after partial hepatectomy. (**A**) Representative immunohistochemistry images of hepatocytes in anaphase of wild type and p38α knockout liver sections after partial hepatectomy. (**B**) Representative immunohistochemistry images of hepatocytes in telophase and cytokinesis of wild type and p38α knock out liver sections after partial hepatectomy. Red, β-tubulin; green, MNK1; blue, DAPI. A minimum of four experiments were performed for each groups of animals. Scale bars = 10 μm. (**C**) Fluorescence intensity of MNK1 staining and quantification of the co-localization of MNK1 and β-tubulin staining in immunohistochemistry images of hepatocytes in anaphase and fluorescence intensity of MNK1 in spindle midzone in cytokinesis images of wild type and p38α knockout liver sections after partial hepatectomy. Data are shown as mean and SD. *P < 0.05; **P < 0.01. WT, wild type mice; KO, p38α knockout mice; PHx, partial hepatectomy.

Immunohistochemistry of PCNA and phospho-H3 (p-H3) in livers of wild type and p38α knockout mice showed a large increase in the mitotic index (calculated as p-H3/PCNA ratio) in p38α knockout animals after PHx (Fig. 2A,B). Moreover we performed western blotting of nuclei isolated from whole livers to detect PCNA and p-H3. As expected, partial hepatectomy was associated with hepatocyte proliferation evidenced by increased PCNA protein levels, which occurred in both wild type and p38α knockout livers (Fig. 2C and Supplementary Fig. S2A). Interestingly, a marked increase in p-H3 occurred only in p38α deficient hepatocytes after PHx but not in wild type hepatocytes (Fig. 2C and Supplementary Fig. S2A). Besides, the lack of p38α in hepatocytes caused an increase in p21 and p53 levels under basal conditions, but after partial hepatectomy these proteins were down-regulated (Fig. 2C and Supplementary Fig. S2A). Our previous findings indicated a failure to complete mitosis due to cytokinesis blockade as the cause of the high mitotic index in p38α deficient hepatocytes^3,10^. Hence, we measured phospho-MNK1 levels, as a critical p38α downstream signaling mediator involved in centriolin localization during mitosis. We found that p38α deficiency decreased phospho-MNK1 levels (Fig. 2D and Supplementary Fig. S2B). Furthermore, the analysis of MNK1 localization by immunofluorescence during the cell cycle revealed the co-localization of MNK1 and tubulin filaments during anaphase in sections from wild type livers, whereas MNK1 was not observed around tubulin filaments in p38α knockout livers (Fig. 3).

### p38α MAPK deficiency triggered a transient delay in liver regeneration after partial hepatectomy

**Figure 4:**
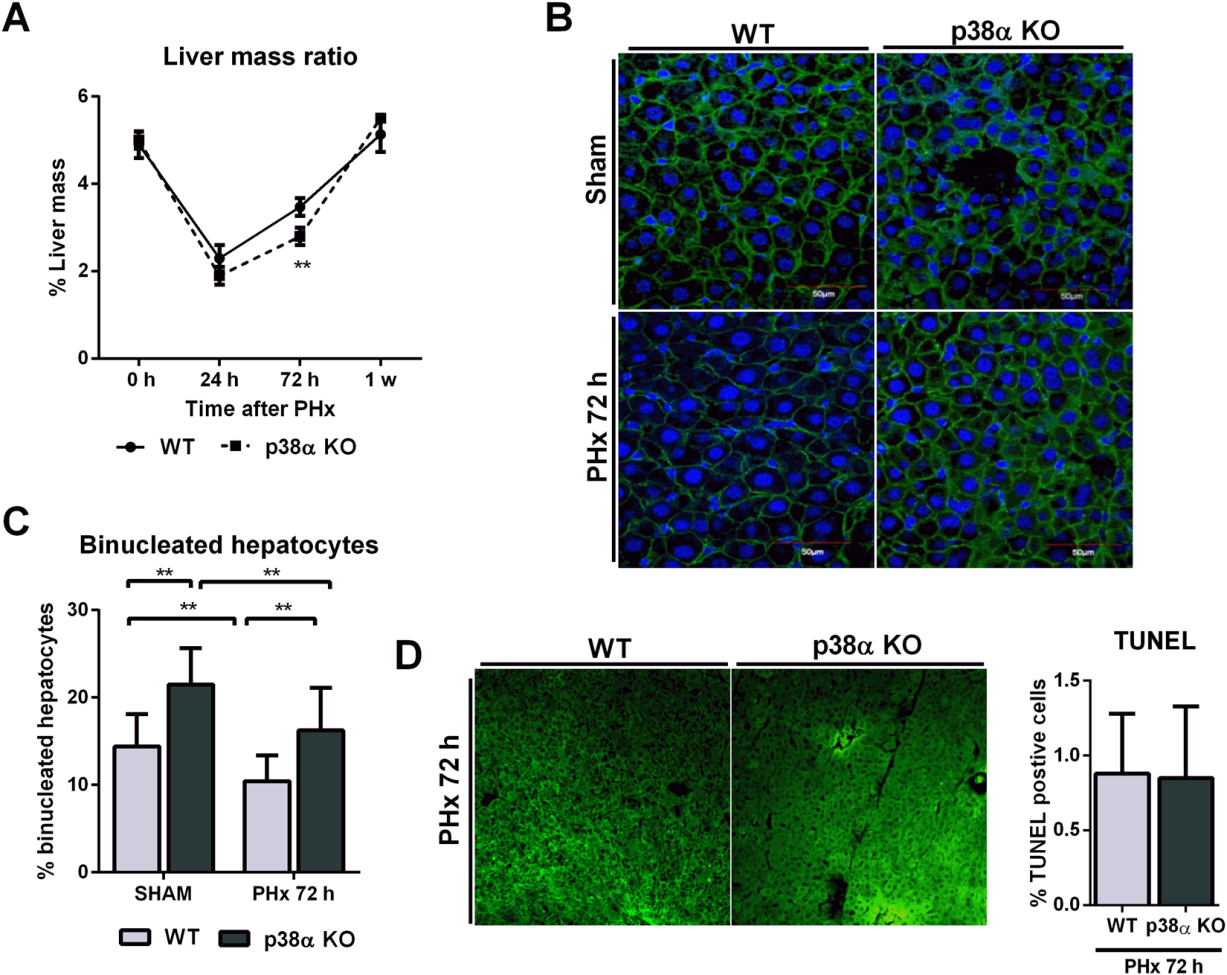
p38α MAPK deficiency restrains liver regeneration after partial hepatectomy and induces hepatocyte binucleation. (**A**) Liver mass ratio, expressed as the ratio: liver weight/body weight (%), in sham wild type and p38α knockout mice 24 h, 72 h and one week after partial hepatectomy. Data are shown as mean and SD. **P < 0.01. (**B**) Representative images of β-catenin (green) and DAPI (blue) immunohistochemistry in sham and 72 hours after PHx of wild type and p38α knockout liver mice. Scale bars = 50 µm. (**C**) Quantification of the binucleation rate (%) (binucleated hepatocytes /total hepatocytes) in sham and after 72 h of PHx based on the immunohistochemistry analysis. At least 20 fields per animal were examined. Data are shown as mean and SD. **P < 0.01. (**D**) Representative TUNEL immunohistochemistry images from wild type and p38α knockout liver sections after PHx (scale bars = 50 µm) and quantification of TUNEL-positive cells expressed as percentage of TUNEL-positive cells/total nuclei. A minimum of four experiments were performed for each group of animals. WT wild type mice; KO p38α knockout mice; PHx partial hepatectomy.

p38α deficiency triggered a transient delay in liver regeneration after partial hepatectomy (Fig. 4A). Thus, at 72 h after partial hepatectomy wild type mice recovered the liver mass to a higher degree compared to p38α knockout mice (Fig. 4A). However, at 1 week after hepatectomy the liver mass in p38α knockout mice was completely recovered as in the control group.

This transient reduction in the recovery of the liver mass was associated with increased binucleation rates in p38α deficient hepatocytes (binucleated hepatocytes/total hepatocytes) (Fig. 4B,C), in accordance with our previous results. However, we did not observe any difference in apoptosis between wild type and p38α knockout livers (Fig. 4D). It is noteworthy that the decrease in binucleation after hepatectomy was observed in both wild type and p38α knockout mice (Fig. 4C).

### p38α MAPK deficiency triggers oxidative stress and liver injury after partial hepatectomy

**Figure 5:**
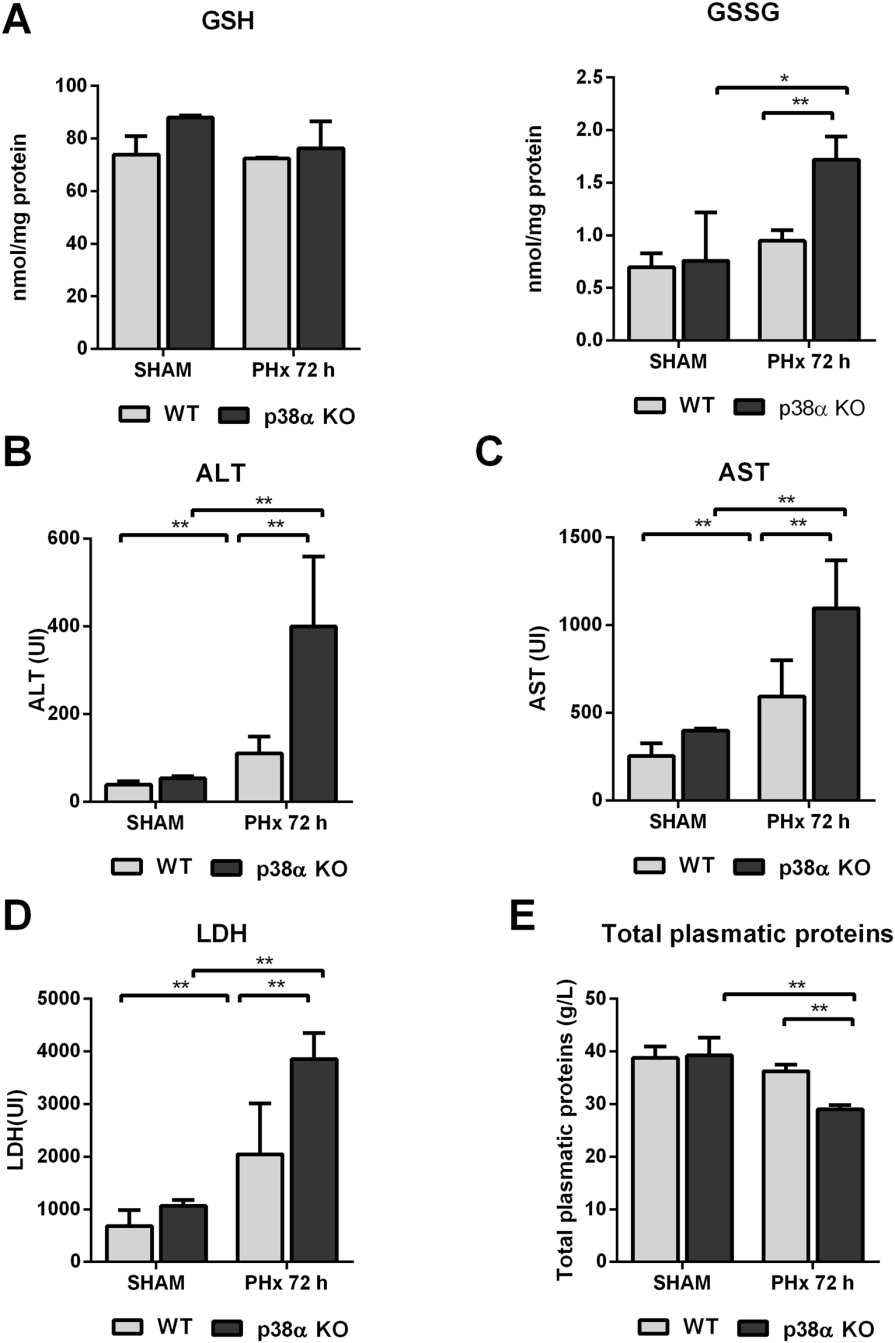
p38α MAPK deficiency triggers oxidative stress and liver injury after partial hepatectomy. (**A**) Hepatic levels of reduced (GSH) and oxidized (GSSG) glutathione. (**B**) Plasma activities of alanine aminotransferase (ALT), (**C**) aspartate aminotransferase (AST) and (**D**) lactate dehydrogenase and (**E**) plasma protein levels in wild type and p38α knockout mouse after partial hepatectomy. Data are shown as mean and SD. *P < 0.05 **P < 0.01. WT wild type mice; KO p38α knockout mice; PHx partial hepatectomy.

In livers of mice with p38α-deficient hepatocytes, we observed that oxidized glutathione (GSSG) levels increased markedly at 72 h after partial hepatectomy (Fig. 5A). In addition, the lack of p38α enhanced liver injury evidenced by the remarkable increase in plasma aminotransferases and lactate dehydrogenase activities found at 72 h after partial hepatectomy (Fig. 5B–D). Furthermore, a decrease in plasma protein levels –a sign of impaired liver function- was observed in mice with p38α-deficient hepatocytes after PHx but not in wild type mice (Fig. 5E).

### p38α MAPK silencing in cultured hepatocytes increases binucleation through blockade of MNK1 phosphorylation

**Figure 6:**
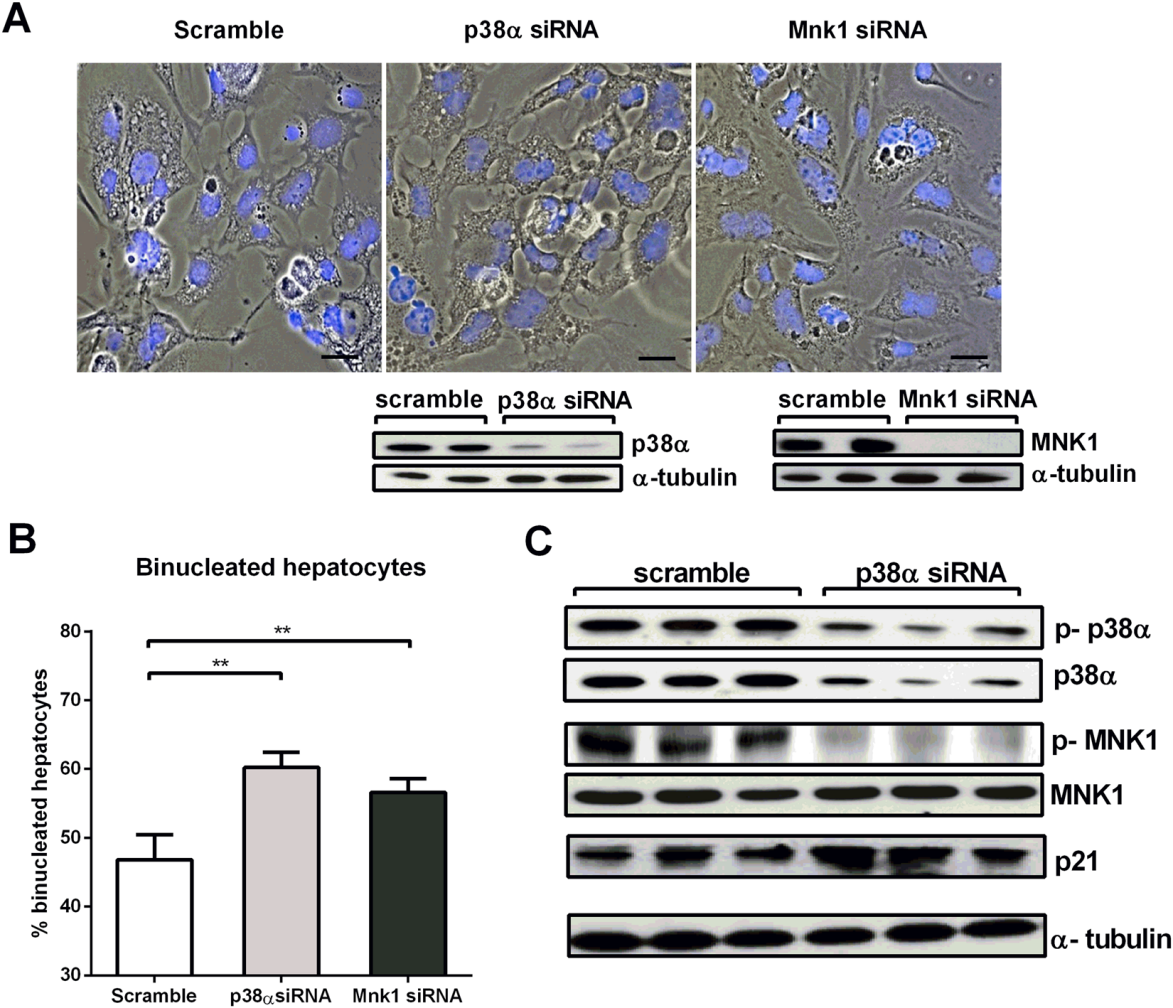
p38α MAPK silencing in primary hepatocytes increases binucleation through blockade of MNK1 phosphorylation. (**A**) Representative images of isolated hepatocytes treated with scramble, *p38α MAPK* siRNA and *Mnk1* siRNA (scale bars = 50 µm). Control for *p38α MAPK* and *Mnk1* silencing by siRNA in isolated hepatocytes is also shown. (**B**) Quantification of the binucleation rate (%) (binucleated hepatocytes/total hepatocytes) in scramble-treated, *p38α MAPK* siRNA-treated and *Mnk1* siRNA-treated primary culture hepatocytes. At least 20 fields per condition were examined. Data are shown as mean and SD. **P < 0.01. (**C**) Western blot of isolated hepatocytes scramble-treated, *p38α MAPK* siRNA-treated and *Mnk1* siRNA-treated for p-p38, p38α, p-MNK1, MNK1 and p21. α-tubulin was used as loading control. The number of samples per group was 4 to 8.

As MNK1 is an important mediator of p38α signaling in the regulation of cytokinesis, we silenced both p38α and Mnk1 in mouse primary hepatocytes (Fig. 6A and Supplementary Fig. S3A). Down-regulation of either p38α or Mnk1 caused a clear increase in the binucleation of cultured hepatocytes (Fig. 6B). Importantly, p38α silencing in primary hepatocytes resulted in a marked decrease in phospho-MNK1 levels, and we also observed the up-regulation of p21 (Fig. 6C and Supplementary Fig. S3B), consistent with the results in mice with p38α-deficient hepatocytes.

## Discussion

Binucleation has its pros and cons. It has some potential advantages, such as gain of function or genetic protection^13^. However it was considered a disadvantage when liver needs to grow^14,15^. Binucleated hepatocytes seem to meet more difficulties when undergoing cell cycle as they have more complexity^14,15^. This hypothesis has been questioned since Duncan and collaborators reported that hepatocytes are able to reverse its ploidy^11^. Ploidy reversal has casted doubt upon classical hepatocyte proliferation principles that considered polyploid hepatocytes as a growing disadvantage. A remarkable feature of liver regeneration after partial hepatectomy in mice is the reduction in the percentage of binuclear polyploid hepatocytes and the increase in the percentage of mononuclear polyploid hepatocytes^16–21^. During liver regeneration after 70% partial hepatectomy, diploid mononuclear, polyploid mononuclear, and polyploid binuclear hepatocytes all may enter the cell cycle leading generally to mononuclear daughter cells, a majority of them polypoid^16–21^. At present it is unknown how highly binucleated hepatocytes face liver regeneration and whether p38α MAPK plays a role in this regard.

Partial hepatectomy is a proliferative stimulus that induces compensatory growth in the liver and represents the most commonly intervention used for studying liver regeneration because it lacks massive necrosis or inflammation^22^. After 5 to 7 days of partial hepatectomy, rodents have usually recovered most of the original liver mass^23^. Intense liver growth takes place on the first 6 days and many mitotic processes are completed by day 3^22^.

Our results show that binucleated p38α-deficient hepatocytes were able to divide into mononuclear hepatocytes in order to efficiently proliferate. Hepatocytes deficient in p38α MAPK from mice could reduce their binucleation rates when a compensatory hepatomegaly after liver resection is needed, although p38α deficient hepatocytes were still more binucleated.

The liver ratio in mice with p38α-deficient hepatocytes was significantly reduced at 3 days after partial hepatectomy compared to those from wild type mice, but at 1 week after hepatectomy the liver mass was completely recovered as in the control group. It has been reported that p38α is rapidly inactivated at 30 min after partial hepatectomy and later on it is re-activated at 12 h after partial hepatectomy^2^. In Supplementary Figs S4 and S5 we show that p38α is re-activated at 24 h after hepatectomy but it is still less active than in controls, and Fig. 2 shows that it is completely reactivated at 72 h after hepatectomy. Hence, the re-activation of p38α between 24 and 72 h after hepatectomy would be required for normal recovery of the liver mass during this period, whereas later on liver regeneration occurs independently from p38α. Indeed, one week after partial hepatectomy p38α phosphorylation is similar to controls and its deficiency does not affect significantly markers of hepatocyte mitosis and proliferation, which are similar to controls (see Supplementary Figs S4 and S5).

The lack of p38α releases the brake on cyclins A1, B1, B2, and D1, but it could be again impairing the last stage of mitosis leading to cytokinesis failure. Accordingly, the high *cyclin A* and *cyclin B* mRNA levels may be due to mitotic arrest. Nevertheless, after hepatectomy it is likely that post-transcriptional regulatory mechanisms of the cell cycle control the protein levels of cyclins B1 and D1, which were similar in p38α deficient mice and wild type mice.

After 72 hours of hepatectomy, p38α knockout hepatocytes displayed higher nuclear levels of phosphorylated H3 in comparison with wild type hepatocytes. Thus, blockade of cytokinesis would trigger the accumulation of hepatocytes in the mitotic stage^24^. In addition, under basal conditions, p38α knockout hepatocytes show higher levels of p21 protein, which has been usually linked to the inhibition of cell cycle progression^25–27^. It is intriguing that the release of one cell cycle brake, such as p38α, results in the up-regulation of another brake, such as p21, which seems to be due to the high p53 levels associated with p38α deficiency. Moreover, the mitogen stimulation associated with partial hepatectomy caused down-regulation of both p53 and p21 in the knockout mice. In fact, the decrease in p21 levels is likely to be due to the reduction of p53 levels upon hepatectomy.

Previous work has reported the requirement of p38α for mitosis entry, proper spindle assembly and checkpoint functions in human, mouse, and rat-kangaroo cells^28^ as well as HCT116 cells^24^, and, in this regard, our results support a similar function for p38α in hepatocytes *in vivo*.

Campbell and collaborators reported that p38 is rapidly inactivated 30 minutes after partial hepatectomy, corresponding with the activation of protein synthesis, and re-activated after 12 h, concluding that p38α activity does not appear to be required for DNA replication *in vivo* during liver regeneration^2^. In agreement with these findings, low p38α MAPK activity was found during embryonic development^5^. Therefore, p38α might not be required for DNA synthesis in hepatocytes, but it certainly regulates mitotic completion and cytokinesis.

Regarding the downstream pathways regulated by p38α that could control cytokinesis, we show that p38α clearly targets MNK1 in the liver. As described by Rannou and collaborators^29^, dephosphorylation of MNK1 leads to the generation of multinucleated cells by deregulating centriolin localization and thus, affecting cytokinesis^30^. In fact, the *Drosophila* Mnk1 ortologue (Lk6) interacts with microtubules and localizes to the centrosomes^31,32^ and it is necessary for cell abscission^29^. We observed reduced MNK1 phosphorylation in p38α-deficient hepatocytes both *in vivo* and in culture, and experiments with isolated hepatocytes demonstrate that MNK1 is responsible, at least in part, for the binucleation induced by p38α deficiency.

In addition, the higher glutathione oxidation observed in p38α-deficient livers after partial hepatectomy could also contribute to the cytokinesis blockade. We previously showed that oxidative stress induces hepatocyte binucleation during the isolation of hepatocytes^33^. The same glutathione redox status was found in both wild type and p38α-deficient livers under basal conditions, but p38α knockout mice subjected to partial hepatectomy exhibited higher glutathione oxidation and consequently more oxidative stress in the liver. Therefore, the impact of oxidative stress on cytokinesis failure during the hepatectomy-induced proliferative response should be considered. Furthermore, although liver regeneration takes place in absence of p38α, it is associated with oxidative stress, tissue injury, and impaired hepatic function.

In conclusion, our results suggest that p38α is necessary for normal hepatocyte mitotic progression and contributes to cytokinesis by activating MNK1 and perhaps also by redox modulation. Its deficiency triggers a transient delay in the recovery of liver mass as well as oxidative stress, tissue injury, and impaired hepatic function after partial hepatectomy.

## Methods

### Animals

p38α MAPK was specifically down-regulated in hepatocytes by using mice carrying *p38α* floxed alleles and the Afp-Cre transgene that expresses Cre under the control of the α-fetoprotein promoter, which is active during embryonic hepatic development. The liver-specific p38α knockout mice were kept in a C57BL/6 genetic background^34^. We performed the partial hepatectomy experiments with wild type and p38α MAPK knockout mice of 4 weeks-old. p38α knockout mice exhibited some abnormalities in the actin cytoskeleton from 8–10 weeks of age as we previously reported^10^. Therefore, we preferred to assess liver regeneration in these mice just after weaning when their actin filaments were well conserved and only the deficiency in MNK1-phosphorylation was likely to affect the completion of cytokinesis in proliferating hepatocytes. Otherwise, the impairment in actin polymerization associated with p38α deficiency that we described in our previous work could have a significant effect on cytokinesis and liver regeneration. For primary hepatocyte cell culture male C57BL/6 mice at 3–4 months of age were used. The *in vitro* experiments were performed in wild type mice at 8 weeks of age to selectively target MNK1 or p38α for binucleation studies in mature livers avoiding the potential bias of mice livers still maturing and undergoing polyploidization at 4 weeks of age and more prone to proliferation upon hepatocyte isolation. Four to eight animals were used for each experimental group in all cases.

All mice received care in accordance with the criteria outlined in the Guide for the Care and Use of Laboratory Animals (NIH publication 86–23 revised 1985) and they were maintained under controlled conditions of temperature (23 ± 1 °C), relative humidity (50–60%) and light/dark cycles (12 h/12 h) with food and water *ad libitum*. The study was approved by the Ethics Committee of Animal Experimentation and Welfare of the University of Valencia (Valencia, Spain).

### Partial hepatectomy

Animals were distributed into eight groups: six groups that underwent partial hepatectomy (PHx wild type and PHx p38α knockout sacrificed at 24, 72 hours or 1 week after PHx), and two groups that were SHAM-operated (SHAM wild type and SHAM p38α knockout). The total number of mice used was 45: 30 animals underwent partial hepatectomy (15 wild type and 15 p38α knockout mice sacrificed after PHx) and 15 sham-operated mice (8 wild type and 7 p38α knockout).

The hepatectomy (PHx) performed in these mice consisted in the resection of 70% of the total liver mass^35^. The mice were operated under anesthesia (3% isoflurane in oxygen) and with the use of a heating mantle at 37 °C. The animal’s abdomen was shaved and disinfected (70% EtOH) to maintain a semi-sterile condition, necessary to reduce the risk of post-operative infections. An incision about 2 cm was made in the abdomen longitudinally along the alba line. Liver sections resected were: right anterior segment, left anterior segment, left posterior segment and omental segment. After suture, the animal was disinfected with iodine tincture and placed on an electric blanket at 37 °C. Both SHAM and PHx animals were treated with analgesia during the entire post-operative period of time (Buprex®). Finally, animals were euthanized under anesthesia at 72 hours post-surgery. Blood was extracted and centrifuged 15 min, 1700 rpm at room temperature to obtain plasma samples, which were frozen at −80 °C till analysis.

### Culture of primary hepatocytes

Primary mouse hepatocytes were isolated from non-fasting male C57BL/6 mice (3–4 months) by a two-step collagenase perfusion as previously described^36^. Briefly, cells were seeded on collagen-coated 12-well plate (Corning, Inc.) and cultured at density of 350,000 cells/well in 1 ml medium containing Dulbecco’s modified Eagle’s medium and Ham’s F-12 medium (1:1) supplemented with 10% FBS, 2 mM glutamine, 100 units/ml penicillin, 100 μg/ml streptomycin, and 1 mM sodium pyruvate (attachment medium) and maintained under a humidity conditions in 95% air and 5% CO_2_ at 37 °C for 24 h before siRNA transfection. For cell staining experiments, collaged-coated coverslips were used.

### Gene knockdown by siRNA

Cells were transfected with 25 nM siRNAs or with a scrambled siRNA, used as control, following DharmaFECT General Transfection Protocol (Dharmacon) to knockdown mouse p38α MAPK and Mnk1 expression. Cells were used 48 h later for experiments. *DharmaFECT siRNA Transfection Reagent*, p38α MAPK siRNA (5 nmol SMARTpool: ON-TARGETplus Mapk14 siRNA L-040125-00-0005), Mnk1 siRNA (5 nmol SMARTpool: ON-TARGETplus Mouse Mknk1 siRNA L-040139-00-0005) and scramble siRNA were obtained from Dharmacon.

### Western blotting

For total liver homogenates, protein was extracted with Heidolph (RZR 2021) homogenizer in Hepes lysis buffer pH 7.4 with 1 mM DTT, 1 mM sodium ortovanadate, 50 mM sodium fluoride, 30 mM sodium pyrophosphate, 1% Igepal, 10% glycerol and protease inhibitor cocktail (Sigma Aldrich). To obtain total cell lysates, attached cells were scraped off and incubated for 10 min on ice with lysis buffer. Homogenates and cell lysates were centrifuged during 15 min at 15000 rpm, 4 °C. In case of nuclei isolation, a slight modification of the nuclei isolation method described by^37^ was used. Chemiluminescence was detected with a charge-coupled device camera (Biorad ChemiDoc XRS+ Molecular Imager and LAS-3000, Fujifilm) using the ECL system (Luminata Classico, Millipore).

Antibodies used were: α tubulin (Sigma Aldrich T6074), p21 (sc-6246), p38α MAPK (sc-535) from Santa Cruz Biotechnology; H3 (4499), MNK1 (2195), PCNA (2586), p53 (9282), p-H3 (Ser10) (9710), p-MNK1(Thr197/202) (2111), p-p38 (Thr 180/Tyr188) (4511XP) from Cell Signaling Technology. All of them were dissolved in 1% Bovine Serum Albumin (Sigma) in TBS-0.01% Tween 20. The blocking agent was a solution of 5% Bovine Serum Albumin in TBS-0.01% Tween 20.

Secondary antibodies were from Jackson Immunoresearch: Donkey anti rabbit (711-035-152) and donkey anti mouse (715-035-151). They were prepared at 1/40,000 dilution in 1% TBS-0.01% Tween 20.

### Real time RT- PCR

A small piece (approximately 30 mg) of liver was homogenized in trizol reagent, and total RNA was isolated by the chloroform:phenol method (Sigma-Aldrich). RT was performed using the Revert Aid kit (Thermo Scientific, Logan, UT). Quantitative PCR experiments were performed on a thermal cycler (I-Cycler + IQ Multicolor Real Time OCR Detection System, Biorad) by using SYBR Green PCR Master Mix (Takara, Otsu, Shiga). Results were normalized using Tbp as reference (mm.tbp.F: CAGCCTTCCACCTTATGCTC; mm.tbp.R: CCGTAAGGCATCATTGGACT). The threshold cycle (Ct) was determined, and relative gene expression levels were subsequently calculated by the 2^(−ΔΔCt)^ formula. Primer sequences used for cyclin expression are shown in Table 1.

**Table 1:** Primer sequences.

Oligo name	Sequence 5′ to 3′
mm.CyclinA1.F	GTCAACCCCGAAAAACTGGC
mm.CyclinA1.R	GAGCAACCCGTCGAGTCTT
mm.CyclinB1.F	GCCTGAACCTGAACTTGAACA
mm.CyclinB1.R	ACATCCAGATGTTTCCATCG
mm.CyclinB2.F	ACCCACAGCCTCTGTGAAAC
mm.CyclinB2.R	CTTGCAGAGCAGAGCATCAG
mm. CyclinCdc25b.F	AGGGAGAGAAGGTGTCTTACCA
mm. CyclinCdc25b.R	GCCCTTTCGACAGGAGTCAA
mm.CyclinD1.F	ACTGCCGAGAAGTTGTGCAT
mm.CyclinD1.R	AAGCAGTTCCATTTGCAGCAG

### Mass spectrometry

Frozen liver samples were homogenized in phosphate saline buffer (PBS) with 10 mM N-ethyl maleimide (NEM). Perchloric acid (PCA) was then added to obtain a concentration of 4% and centrifuged at 15,000 g for 15 min at 4 °C. The concentrations of GSH and GSSG were determined in the supernatants by high-performance liquid chromatography coupled to tandem mass spectrometry (HPLC-MS/MS). The chromatographic system consisted of a Micromass QuatroTM triplequadrupole mass spectrometer (Micromass, Manchester, UK) equipped with a Zspray electrospray ionization source operating in the positive ion mode with a LC-10A Shimadzu (Shimadzu, Kyoto, Japan) coupled to the MassLynx software 4.1 for data acquisition and processing. Samples were analyzed by reverse-phase HPLC with a C18 Mediterranea SEA column (Teknokroma, Barcelona, Spain). The mobile phase consisted of the following gradient system (min/%A/%B) (A, 0.5% formic acid; B, isopropanol/acetonitrile 50/50; 0.5% formic acid): 5/100/0, 10/0/100, 15/0/100, 15.10/100/0, and 60/100/0. The flow rate was set at 0.2 ml/min. Positive ion electrospray tandem mass spectra were recorded using the following conditions: capillary voltage 3.5 kV, source temperature 120 °C, nebulization and cone gases were set at 500 and 30 L/h, respectively. Argon at 1.5610–3 mbar was used as the collision gas for collision-induced dissociation. An assay based on LC-MS/MS with multiple reaction monitoring was developed using the transitions m/z, cone energy (V), collision energy (eV) and retention time (min) for each compound that represents favorable fragmentation pathways for these protonated molecules (Table 2). Calibration curves were obtained using twelve-point (0.01–100 mmol/l) standards (purchased from Sigma-Aldrich, St Louis, USA) for each compound. The concentrations of metabolites were expressed as nmol/mg of protein.

**Table 2:** Transitions and retention times for analytes determined by LC–MS/MS.

Analyte	Cone (V)	Collision (eV)	Transition (*m*/*Z*)	Retention time (min)
GS-NEM	30	15	433 > 304	4.32
GSSG	30	25	613 > 355	1.46

### Immunohistochemistry and cell staining

Formalin-fixed, paraffin embedded sections of liver tissue were deparaffinized using Histo-Clear® (National Diagnostics). As an alternative for xylene and antigen retrieval, tissue sections were autoclaved in citrate buffer pH 6 for staining. Slides were blocked with 5% BSA in PBS. Antibodies used in immunofluorescence were as follows: β-catenin (9562), Mnk1 (Thr197/202) (2111), p-H3 (ser10) (9710) and PCNA (2586) from Cell Signaling Technology and β-tubulin (ab7792) from Abcam. All of them were dissolved in 1% Bovine Serum Albumin (Sigma) in TBS-0.3% Tween 20 and purchased from Cell Signaling Technology. Secondary antibodies were: Alexa Fluor anti rabbit (A-21206) and Alexa Fluor anti mouse (A-11002) from Life Technologies. Nuclei were stained with DAPI (Life Technologies, D1306). For binucleation rate (percentage of binucleated cells/total number of cells) and number of nuclei per field, 50–60 slides from all different animals were blindly scored.

For *in vitro* assays, primary hepatocytes seeded on collagen-coated glass coverslips (10 mm) in 12-well plates were fixed with 4% paraformaldehyde for 6 min, washed with PBS and permeabilized with 0,1% Triton X-100 during 5 min. Nuclei were stained with DAPI (Life Technologies, D1306). An OLYMPUS FV1000MPE confocal microscope was used for image acquisition.

### Biochemical assays

Liver injury and function was assessed by alanine aminotransferase (ALT, Spinreact 41280), aspartato aminotransferase (AST, Spinreact 41270), and lactate dehydrogenase (LDH, Spinreact 1001260) activities in plasma. Moreover, total plasmatic proteins (Spinreact 1001290) were determined. Plasma samples were analyzed according to the manufacture instructions.

### TUNEL assay

Formalin-fixed, paraffin embedded sections were deparaffinized using xylene and then, antigen retrieval was performed by citrate buffer (37 °C, 15 minutes). Nucleotide labelling and detection were performed as described following manufacturer’s instructions (*In Situ* Cell Death Detection Kit 11 684 817 910, Roche).

### Statistical analysis

Results are given as mean ± standard deviation (s.d.). Significant differences were assessed by one-way analysis of variance (ANOVA) followed by a Tukey’s *post-hoc* test. Differences were considered statistically significant at p < 0.05.

## Supplementary information

Revised Supplementary Info
