# Supplementary material for: p38α deficiency restrains liver regeneration after partial hepatectomy triggering oxidative stress and liver injury
Source: Sci Rep. 2019 Mar 7;9:3775. doi: 10.1038/s41598-019-39428-3 (PMC6405944; doi:10.1038/s41598-019-39428-3)
Supplement: Supplementary file 1 — Revised Supplementary Info [file 41598_2019_39428_MOESM1_ESM.pdf]

# p38α deficiency restrains liver regeneration after partial hepatectomy triggering oxidative stress and liver injury

**Sergio Rius-Pérez[1], Ana M. Tormos[1], Salvador Pérez[1], Isabela Finamor[1], Patricia Rada[2,3], Ángela M. Valverde[2,3], Angel R. Nebreda[4,5], Juan Sastre[1] and Raquel Taléns-Visconti[6*]**

[1] Department of Physiology, University of Valencia. Burjassot, Valencia, 46100 Spain.

[2] Instituto de Investigaciones Biomédicas Alberto Sols (Centro Mixto CSIC-UAM), Arturo Duperier 4, 28029 Madrid, Spain.

[3]Centro de Investigación Biomédica en Red de Diabetes y Enfermedades Metabólicas Asociadas (CIBERdem), ISCIII, 28029 Madrid, Spain.

[4]Institute for Research in Biomedicine (IRB Barcelona), Barcelona Institute of Science and Technology, 08028 Barcelona, Spain.

[5]ICREA, Pg. Lluís Companys 23, 08010 Barcelona, Spain.

[6]Department of Pharmacy and Pharmaceutical Technology and Parasitology, University of Valencia. Burjassot, Valencia, 46100 Spain.

***Corresponding author:**

E-mail: raquel.talens@uv.es

# Supplemental Figure S1

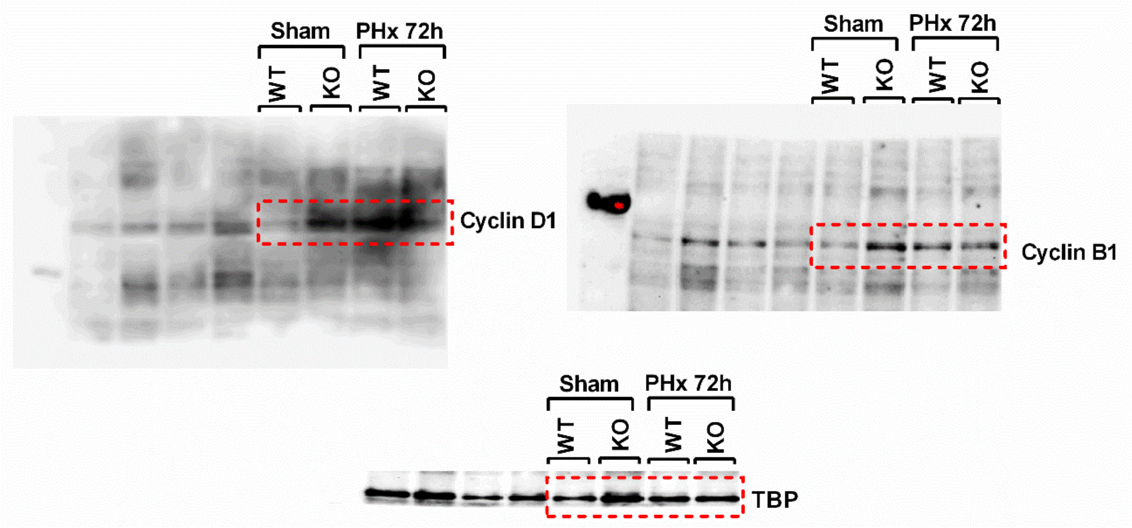


**Figure S1.** Full length blots of Figure 1b. Red dotted lines show the cropping area.

# Supplemental Figure S2

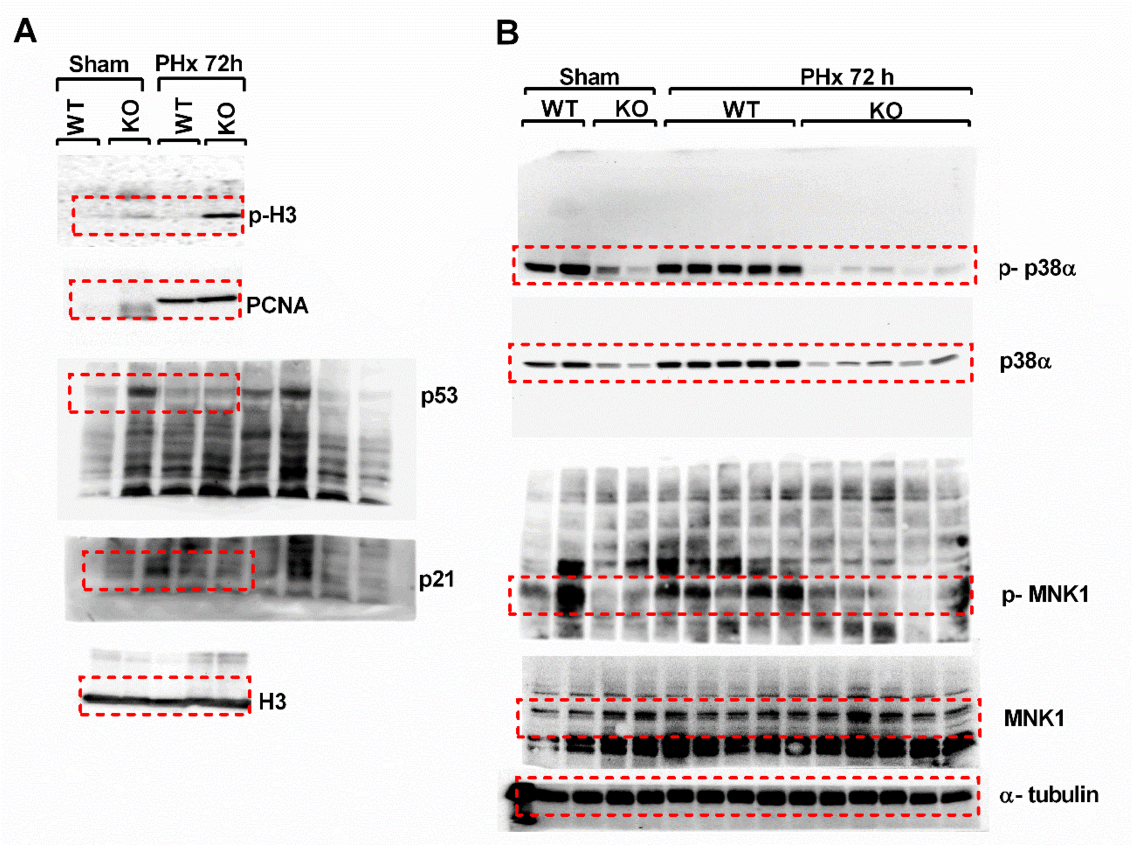


**Figure S2. (a)** Full length blots of Figure 2c. Red dotted lines show the cropping area.

(**b**) Full length blots of Figure 2d. Red dotted lines show the cropping area.

# Supplemental Figure S3

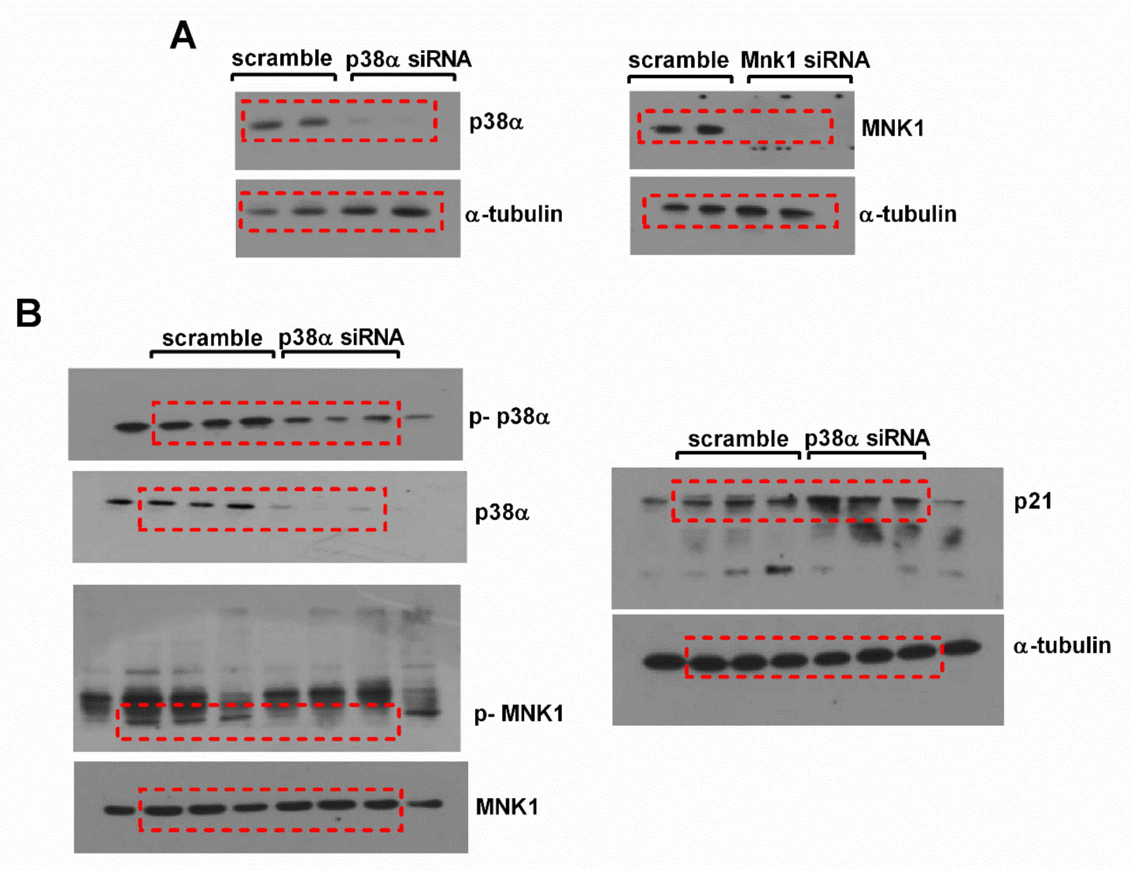


**Figure S3. (a)** Full length blots of Figure 6a. Red dotted lines show the cropping area.

**(b)** Full length blots of Figure 6c. Red dotted lines show the cropping area.

# Supplemental Figure S4

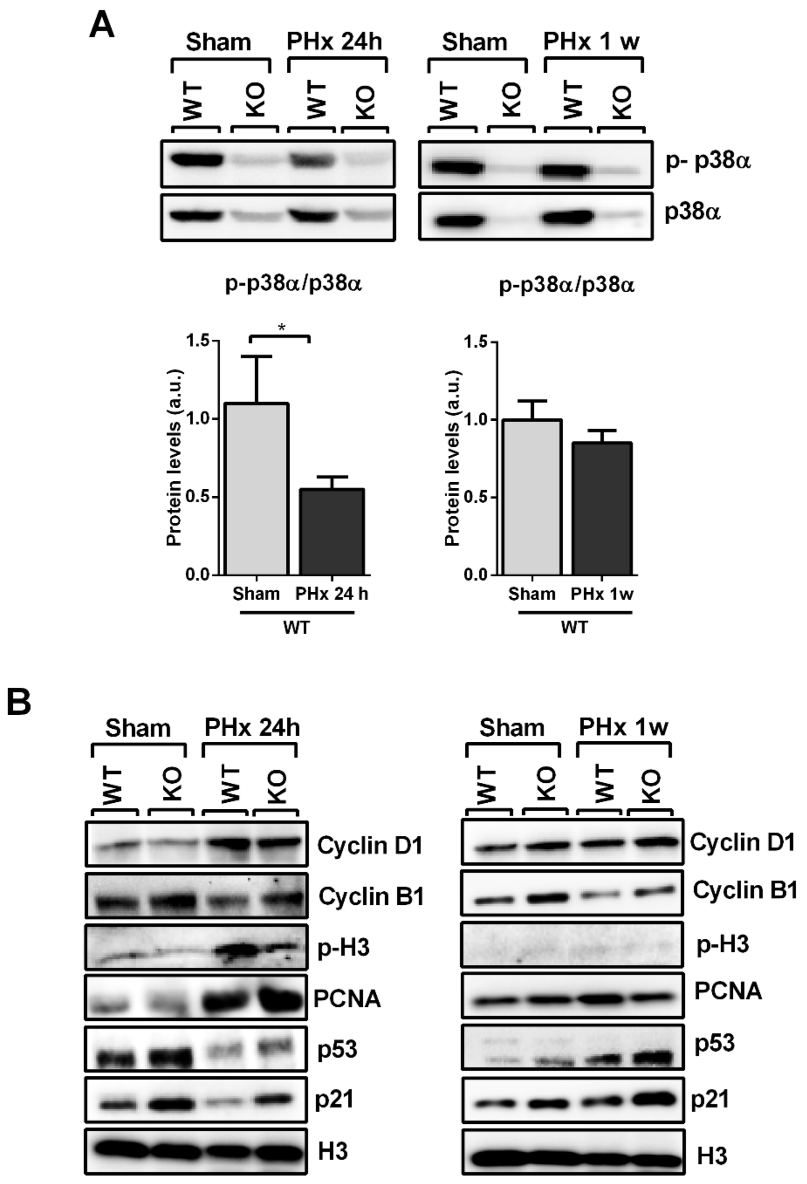


**Figure S4. (a)** Representative western blot images and densitometries for p-p38α and p38α of livers from sham and 24 hours and 1 week after PHx. **(b)** Representative western blot for cyclin D1, cyclin B1, p-H3, PCNA, p53, p21 and H3 in nuclear extracts of livers from sham wild type and p38α knockout mice and at 24 hours and 1 week after PHx. H3 was used as loading control. *P < 0.05. WT, wild type mice; KO, p38α knockout mice; PHx, partial hepatectomy.

# Supplemental Figure S5

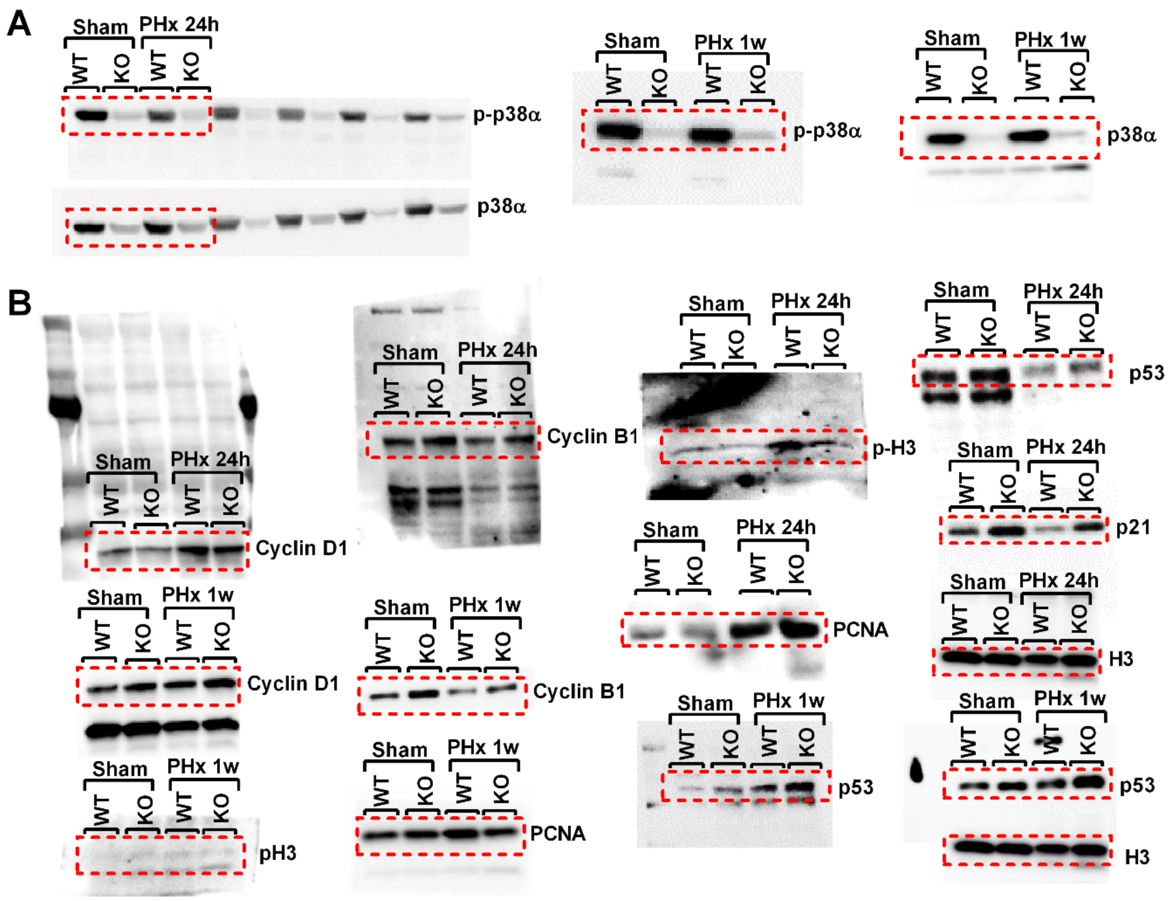


**Figure S5. (a)** Full length blots of Figure S4a. Red dotted lines show the cropping area.

**(b)** Full length blots of Figure S4b. Red dotted lines show the cropping area.
